# Indicators Developed to Evaluate the International Framework Convention on Tobacco Control in Iran; A Grounded Theory Study

**Published:** 2014-03

**Authors:** Nizal Sarrafzadegan, Katayoun Rabiei, Heidarali Abedi, Roya Kelishadi, Khadijeh Fereydoun Mohaseli, Mousa Alavi, Hamidreza Roohafza

**Affiliations:** 1 Cardiovascular Research Center, Isfahan Cardiovascular Research Institute, Isfahan University of Medical Sciences, Isfahan, Iran;; 2 Rehabilitation Research Center, Isfahan Cardiovascular Research Institute, Isfahan University of Medical Sciences, Isfahan, Iran;; 3 Department of Nursing, Faculty of Nursing and Midwifery, Khorasgan (Isfahan) Branch, Islamic Azad University, Isfahan, Iran;; 4 Director, National Tobacco Control Secretariat, Ministry of Health, Tehran, Iran;; 5 Nursing Research Center, Isfahan University of Medical Sciences, Isfahan, Iran

**Keywords:** Program evaluation, Tobacco, Iran

## Abstract

This study aimed to develop indicators for evaluating the implementation of The Framework Convention on Tobacco Control (FCTC) in Iran. We used the “grounded theory” framework. Totally, 265 policy-makers, stakeholders, and community members were recruited by purposeful sampling in 2008. After analyzing the gathered data, 251 indicators, including 82 indicators as “applied indicators”, were derived from second-level codes for three groups. A suitable evaluation questionnaire can be designed based on the extracted indicators for policy makers, stakeholders, and the community to follow the implementation of the FCTC in Iran.

## Introduction

In 2003, The World Health Organization (WHO) developed The Framework Convention on Tobacco Control (FCTC).^1^ The treaty was discussed and adopted by the 56^th^ World Health Assembly.^1^ Coming into force on February 27, 2005, the FCTC was signed by 168 countries.^2^

Enforcement and implementation of the FCTC articles and assessment of its outcome requires a specifically designed system of evaluation. Hence, the WHO designed a questionnaire to evaluate the enforcement of the FCTC at the country level.^3^ This questionnaire is brief and mainly concerns the implementation of the FCTC policies. It is usually completed by the Ministry of Health authorities.^3^ The largest ongoing international multicentric study to evaluate the impact of the FCTC is The International Tobacco Control Policy Evaluation (ITC). The ITC is a collection of prospective cohort surveys in more than 20 countries to evaluate the impact and identify the determinants of effective tobacco control policies.^4^

Iran has also ratified the FCTC and designed The National Comprehensive Tobacco Control Program (NCTCP).^5^ The implementation of the FCTC in Iran is currently evaluated by the WHO monitoring questionnaire, and all the questions are answered by the Ministry of Health authorities.^3^

Given the cultural and socioeconomic differences between countries and populations and the paucity and insufficiency of the existing evaluation tools, we decided to develop process, impact, and outcome indicators based on our social mores and beliefs to evaluate the implementation of the FCTC. 

## Materials and Methods

Initially, a scientific committee was formed. Then, a literature review of the FCTC evaluation programs or studies was conducted.  Also, all existing documents and circulars in Iran regarding the NCTCP and FCTC objectives were gathered. 

Indicators were obtained through a qualitative study designed on the basis of the “grounded theory”.^6^ Participant selection was done based on people's experiences about developing or implementing tobacco control legislations as policy-makers, tobacco selling and its profits as beneficiaries, and the community as a whole. To that end, three major groups were purposefully selected: policy-makers, stakeholders, and community members. The sample size was determined through data saturation (i.e., sampling until informational redundancy or saturation was achieved).^7^

Field observation and semi-structured individual interviews were done. Primary indicators were extracted from literature review and the NCTCP, and enforcement instructions were used to develop the general questions. 

All interviews were in-depth semi-structured and were done with the interviewees' oral consent. Data collection was performed by the Glaser and Strauss approach.^8^ The useful units of meaning in the interview transcripts were used as first-level codes. The concepts of the first level codes were determined and after merging some similar concepts, second-level codes were derived.^7^ "Evaluation indicators" were defined for all types of evaluation (i.e., impact, process, and outcome) based on the second-level codes. Thereafter, a scientific committee was convened to review the results, finalize the evaluation indicators, and select some as “applied evaluation indicators”. The committee members recommended that the “applied indicators” be considered in the evaluation questionnaire.

## Results

Thirteen policy-makers, 76 stakeholders, and 146 community members were interviewed. 

A total of 617 first-level codes and 251 second-level codes were extracted. All the second-level codes were used to synthesize indicators. After obtaining the committee members’ opinions, 82 indicators were designated as “applied indicators”. 

tables 1, 2 and 3 show the "applied indicators" according to the target group and type of indicators. As is shown in table 1, we extracted 24 indicators for policy-makers' evaluation, in which 2, 3, and 19 indicators belong to outcome, impact, and process evaluation, respectively. From the 27 indicators for stakeholders’ evaluation, 2 indicators evaluate outcomes, 10 indicators evaluate impact, and 15 indicators evaluate the implementation process of the FCTC. Unlike policy-makers and stakeholders, in the community study, most of the 31 indicators belong to impact evaluation (20 indicators) (table 3).

**Table 1:** Applied indicators used to evaluate tobacco control regulations in policy-makers

**Indicators**
Outcome:
Cost-effectiveness of law enforcement
Burden of tobacco-related diseases
Impact:
Reasons for tobacco use in the community
Private sector’s attitude to customers
Contradictions in the state (production as a stakeholder versus control as policy-maker)
Process:
Take penalties
Law prohibiting smoking in public places
Write-off the false labeling of tobacco products
Insert health warnings on the tobacco package
Rules on no sales to minor**s**
Unfavorable media advertisement
Law enforcement
Status of intersectoral collaboration
Financial resources for tobacco control education
News dissemination by media
Monitoring and controlling system
Legal procedures for teahouse licensure
Annual imports
Annual taxes received
Anti-smuggling legislation
Annual tobacco production
Annual tobacco cultivation
Written instructions for enforcement of the laws
New job creation for workers in jobs associated with tobacco

**Table 2:** Applied indicators used to evaluate tobacco control regulations in stakeholders

**Indicators**
Outcome
Quality of life
Change job
Impact
Attitude to old-time tobacco-related business
Social aspects of teahouses and hookah cafes
Cultural changes
Considering tobacco use as a social anomaly
Customers’ attitude to passive smoking
Personal practice of teahouse owners toward tobacco
Rate of dissatisfaction with enforcement of the law
Presence of economic crisis
Attitude toward revenue creation
Extent of stakeholders' support of the law
Process
Advertising the status of the tobacco company
Number of hookah-providing teahouses
Tobacco sales without health warning labels
Impossibility of working without a license
Presence of supervision
Frequency of illegal consumption of the hookah in public places
Prohibition of tobacco use for women
Instructions to persuade customers
The union’s performance toward law enforcement
Amount of tobacco produced by the government
Possibility of farming a different products instead of tobacco
Awareness of the prohibition of tobacco advertising
Type of cigarettes favored by customers
Sale of cigarettes with holograms
Elimination of free promotional tobacco goods

**Table 3:** Applied indicators used to evaluate tobacco control regulations in the community

**Indicators**
Outcome
Treatment and cessation rate
Incidence of tobacco-related diseases
Quality of life
Impact
Attitude of adults toward the effect of advertising on the prevention of use
Attitude toward the necessity of prevention
Youths’ attitude toward growing up with cigarettes
Parents’ attitude toward the presence of cigarette smokers among friends
Attitude toward bad mouth odor and bad appearance of cigarette smokers
Attitude toward the hookah
Adolescents’ eagerness to try
Smoking for pleasure
Effect of family on the students’ picking up cigarette smoking
Negative attitude toward policy involvement/position of police
Degree of belief in the effectiveness of actions taken
Re-smoking following a stressful event
Empowerment of cigarette smokers to cease smoking
Effect of cigarette advertising
Degree of solving adolescents’ problems
Degree of replacing cigarettes with healthy pastimes
Community’s approval of tobacco control laws
Effect of holograms
Effectiveness of messages depicting hazards of smoking
Belief toward media messages
Process
Amount of control exercised by the police
Participation of health sectors in education and training planning and implementation
Participation of municipalities in education and training planning and implementation
Awareness of the prohibition of selling cigarettes to the minor
Awareness of the prohibition of tobacco use in public buildings
Accessibility of cigarettes
Social and mental status of smokers
Productivity of smokers

## Discussion

The government and policy-makers in Iran are responsible not only for tobacco control but also for its production, import, pricing, and taxation. They are also in charge of determining the size and type of warning labels, tobacco advertising prohibition, and supervision of stores, teashops, cafes, and restaurants. Consequently, the mentioned evaluation indices are mainly related to the performance of policy-makers. Nevertheless, considering the role of stakeholders in tobacco industry, measures taken by the government may fail without improving the stakeholders’ knowledge, attitude, and performance along with reducing their benefits. For instance, the emergency plan of the government to collect hookahs faced the vast opposition of teashop owners.    

Creating employment is an issue of high priority to the Iranian government and a factor of crucial significance in the implementation of the NCTCP. One of the indicators that emerged from the interviews was the creation of alternative employment opportunities for people whose livelihoods are associated with tobacco production, supply, and distribution. 

Another important indicator is the control of tobacco smuggling. Annually, 60 billion cigarettes are smoked in Iran.^9^ One-third of these cigarettes are smuggled.^10^ Policies and strategies concerning tobacco smuggling can influence the accessibility of tobacco products. As is shown in table 3, the community attitudes can reduce the demand for tobacco and it is as an essential indicator to tobacco control.

The community’s perception of tobacco control policies can influence the implementation and outcome of such policies; hence, an understanding of the social context where tobacco control policies are to be put into action is an essential component of models for the implementation and evaluation of tobacco control programs.^11^^,^^12^

Similar to ITC evaluation indicator,^13^ impact and outcome indicators are the  important indicators at the community level. Community knowledge, attitude, and practice are the impact indicators. The frequency of quitting tobacco and undergoing withdrawal treatment, the incidence of tobacco-related diseases, morbidity and mortality, and people’s quality of life are examples of outcome indicators.

One of the major problems in Iran and other Middle Eastern countries is hookah smoking as a recreational activity. Therefore, if the public attitude toward hookah smoking does not change, implementing the rule of banning the hookah and removing it from teahouses will be difficult. Therefore, the number of teahouses offering hookahs is an important indicator for evaluating the changes occurring in the stakeholders’ domain. The KAP of parents and their children toward hookah and the prevalence of hookah smoking in the general population, are important impact and outcome indicators for evaluating the implementation of the NCTCP. 

## Conclusion

Evaluation tools for each three target groups should be designed to accommodate all three levels of evaluation and be guided by tobacco control conventions and nationally tailored indicators. As our results demonstrate, we extracted 82 "applied indicators" that comprised all levels of evaluation in the three target groups.
